# Different Clinical Utility of Oropharyngeal Bacterial Screening prior to Percutaneous Endoscopic Gastrostomy in Oncological and Neurological Patients

**DOI:** 10.1155/2014/590891

**Published:** 2014-08-27

**Authors:** Radek Kroupa, Jana Jurankova, Milan Dastych, Michal Senkyrik, Tomas Pavlik, Jitka Prokesova, Marketa Jecmenova, Jiri Dolina, Ales Hep

**Affiliations:** ^1^Department of Internal Medicine and Gastroenterology, University Hospital and Faculty of Medicine, Masaryk University Brno, Jihlavska 20, 625 00 Brno, Czech Republic; ^2^Department of Clinical Microbiology, University Hospital, 625 00 Brno, Czech Republic; ^3^Department of Laboratory Methods, Faculty of Medicine, Masaryk University Brno, 625 00 Brno, Czech Republic; ^4^Institute of Biostatistics and Analyses, Faculty of Medicine, Masaryk University, 625 00 Brno, Czech Republic

## Abstract

*Background*. The aim of this study was to monitor oropharyngeal bacterial colonization in patients indicated for percutaneous endoscopic gastronomy (PEG). *Methods*. Oropharyngeal swabs were obtained from patients prior to PEG placement. A development of peristomal infection was evaluated. The analysis of oropharyngeal and peristomal site pathogens was done. *Results*. Consecutive 274 patients referred for PEG due to neurological disorder or cancer completed the study. Oropharyngeal colonization with pathogens was observed in 69% (190/274), dominantly in the neurologic subgroup of patients (*P* < 0.001). Peristomal infection occurred in 30 (10.9%) of patients and in 57% of them the correlation between oropharyngeal and peristomal agents was present. The presence of oropharyngeal pathogens was assessed as an important risk factor for the development of peristomal infection only in oncological patients (OR = 8.33, 95% CI: 1.66–41.76). Despite a high prevalence of pathogens in neurological patients, it did not influence the risk of peristomal infection with the exception for methicillin resistant *Staphylococcus aureus* (MRSA) carriers (OR 4.5, 95% CI: 1.08–18.76). *Conclusion*. During oropharyngeal microbial screening prior to the PEG insertion, the detection of pathogens may be a marker of the increased risk of peristomal infection in cancer patients only. In neurological patients the benefit of the screening is limited to the detection of MRSA carriers.

## 1. Introduction

Percutaneous endoscopic gastrostomy (PEG) is a widely used method to provide long-term enteral nutrition in patients unable to swallow due to a variety of reasons. Central nervous system (CNS) disorders, for example, stroke or motor neuron disease and head and neck cancer, are typical indications.

PEG tube placement is performed by almost all endoscopic centers. The pull method is a more widely used technique of the procedure due to its simplicity [1]. Patients receiving a PEG catheter are often in poor health and are vulnerable as a consequence of comorbidities and malnutrition [2]. The infection of the PEG insertion site is still quite a common complication, regarding 4–30% of the patients [3, 4]. Many randomized controlled trials and meta-analyses proved the substantial decrease of wound infections when intravenous or oral antibiotic prophylaxis was given before the procedure [5–8].

The role of the oropharyngeal pathogens was empirically assumed and penicillin-based or cephalosporin-based prophylaxis is generally used. The clinical guidelines of endoscopic societies recommend the use of antibiotic prophylaxis before PEG tube placement in all patients [9–11]. However, the results of many studies show significant discrepancies in clinical outcome and incidence of infection especially between drug and placebo arms.

Peristomal infection may be caused by flora colonizing the upper part of the digestive tube through the transport of microbes to the PEG channel in the abdominal wall. Some studies have shown the emergent increase of the poly resistant pathogens like methicillin resistant* Staphylococcus aureus* (MRSA) in PEG patients [12]. The empirical choice of the proper antibiotics may be ineffective in some patients and may lead to increased infections caused by pathogens resistant to standard prophylaxis. There is low evidence regarding different pathogens in different subgroups of patients modifying the selection of antimicrobial drug.

The aim of our study was to monitor oropharyngeal and airway colonization prior to PEG placement, to determine its relationship to PEG site infection, propriety of used antibiotic prophylaxis, and analysis among different patient populations.

## 2. Methods

A prospective cohort study of consecutive patients with PEG insertion was designed at Department of Internal Medicine and Gastroenterology of University Hospital and Faculty of Medicine, Masaryk University Brno, Czech Republic, over a 60-month period from June 2007 to June 2012. All appropriate patients referred for PEG placement were included. None PEG procedure was done in patients with signs of active infection (fever and/or elevated markers of inflammation). The procedure was conducted after informed consent was given by the patient or his or her guardian. The study was approved by local ethical committee.

### 2.1. PEG Procedure

An oropharyngeal swab was done prior to the endoscopy procedure in the morning. PEG placement was performed under mild sedation using intravenous midazolam 1.5–3 mg. In case of the presence of tracheostoma, material from the sputum was obtained as well. Antibiotic prophylaxis using coamoxicillin 1.2 g intravenously in single dose, 30 minutes before the procedure, was performed. In patients with known allergy to penicillin, cephalosporin-cefuroxime was used. If the patients had already used antibiotic for other diseases, the antibiotic would be kept and attributed as prophylactic antibiotics for PEG.

Standard esophagogastroduodenoscopy was performed in all patients before PEG placement. After transillumination of the abdominal wall PEG catheter Flocare Ch18 (Nutricia) was inserted by standard pull method. Local alcohol based disinfectant was used on skin surface before needle puncture. After procedure, the abdominal PEG site was covered by sterile covering and changed every 24 hours. PEG site was evaluated within 7 days after insertion depending on clinical status of patient and wound referred by caregivers. Signs of erythema, infiltration, induration, exudates of fluid, or pus were interpreted as indicators of the possible presence of the infection. Fever alone without any of the previously named signs was not recorded as an infection of PEG. If suspect, a wound swab culture was obtained from the PEG site. The peristomal infection was defined as a presence of the clinical signs together with positive bacterial culture from the PEG site exudate. Profuse purulent secretion on the abdominal wall and signs of systemic infection and/or pathologic finding on image modalities were considered as a major complication (abscess or phlegmon). Other signs of infection were evaluated as a minor complication.

Mortality within 30 days was also recorded.

As potential risk factors for PEG wound infection these factors were evaluated: sex, age, indication, microbial agents in oropharynx (commensal flora versus pathogens, polymicrobial versus single, amoxicillin sensitive versus resistant, MRSA (methicillin resistant* Staphylococcus aureus*) infection), presence of other complications (e.g., bleeding), presence of serious comorbidities (diabetes, renal failure, severe malnutrition, and liver cirrhosis), and presence of tracheostoma. Identical factors were evaluated in view of risk of 30-day mortality.

### 2.2. Microbiology Evaluation

Evaluation of oropharyngeal, airway swab, and peristomal wound was made by standard methodology. Bacterial cultures were then specified by biochemical tests. Screening for methicillin resistance in* Staphylococcus aureus* and for extended-spectrum beta-lactamase (ESBL) in gram negative bacteria was performed. In vitro desk tests for antibiotic susceptibility were done in pathogenic strains.

The isolates from sputum or oropharynx and peristomal wound in the same patient were labeled as “different” or “similar.” We stated that pathogens are similar if in vitro susceptibility of the same bacterial strain to standard set of tested antibiotic was completely the same. If any difference appeared, the results were determined as different or not concordant.

### 2.3. Statistical Analysis

Standard descriptive statistics were used to summarize patient characteristics. Differences in patient characteristics according to PEG indication were assessed using Fisher exact test. Univariate as well as multiple logistic regression models were used to quantify the influence of individual variables on the PEG site infection. Resulting odds ratios were accompanied with 95% confidence intervals. Correction for multiple testing was not applied due to exploratory nature of this study. Standard 5% level for the statistical significance was considered. Data analysis was performed using SPSS software (SPSS Inc., version 19, Chicago, IL).

## 3. Results

The PEG was inserted in 373 patients (mean age 64.1 ± 16.5 years; 254 male) during the study period. The swab tests prior to PEG insertion were lacking in 68 patients and 31 patients were lost for appropriate follow up. Remaining 274 patients (73%) completed the study and were further analyzed. Summary of patient characteristics according to indication for PEG is listed in Table 1. Neurological disorder was underlying disease for PEG in 206 patients and head and neck cancer in 68 patients. Oropharyngeal colonization with pathogens was observed in 69% (189/274) patients. In vitro resistance of oropharyngeal pathogens to coamoxicillin was observed in 38%. According to corresponding *P* values, statistically significant difference between the two PEG indication groups was found in the frequency of colonizing pathogens, polymicrobial flora, resistance of pathogens to amoxicillin, tracheostomy, other complications, and 30 days of mortality. All of these factors were more prevalent in patients with neurological disorders. Major complications (bleeding from upper GI tract and oropharynx and respiratory problems) occurred more frequently in cancer patients.

Methicillin resistant* Staphylococcus aureus* was observed in 11 patients (4%), all with a neurologic disorder. Resistant agents were dominant in patients indicated due to CNS disorder versus tumors. (45% versus 17% *P* < 0.001). The organisms cultured from oropharyngeal swabs are summarized in Table 2.

### 3.1. The Peristomal Wound Infection

The peristomal infection within 7 days after insertion occurred in 30/274 (10.9%) patients. Severe infection requiring surgery (2) and/or endoscopic PEG removal (3) occurring in 5 patients indicated neurologic disorder. These severe complications were caused by coamoxicillin resistant pathogens. In 29 patients, other noninfectious complications were encountered. Bleeding from gastric mucosa, abdominal wall, pharyngeal or oral tumor mass, diarrhea, constipation, flatulence, and leakage around the catheter were recorded.

Patients with PEG site infection were studied as to whether the oropharyngeal colonizing pathogens could be related to the development of infectious complication. The characteristics of patients with PEG infection are presented in Table 3.

Concordance between oropharyngeal and peristomal microbial agents was 57% (17/30). In 13 other patients pathogens isolated from the PEG site wound were different from colonizing.

There were coamoxicillin resistant agents responsible for PEG site infection in 15 (50%) patients. A summary of microbial agents isolated from wound cultures is given in Table 2.

An infection complication developed in 3 out of the 11 neurological patients colonized by MRSA.

The factors that could enhance the development of the peristomal infection were analyzed in 274 PEG patients. Diagnosis, colonization by pathogens, resistant agents, MRSA, tracheotomy, polymicrobial flora, serious comorbidities, and sex were considered as risk factors. A nonstatistically significant trend towards higher risk of PEG wound infection was observed in cancer patients, OR 2.24 (95% CI: 0.95–5.26, *P* = 0.070). An overview of risk factor analysis is presented in Table 4.

The detailed analysis of possible risk factors with respect to PEG site infection in patients with head and neck cancer is presented in Table 4. In this case, only the presence of colonizing pathogens was found to be statistically significantly associated with PEG site infection (OR = 8.33, 95% CI: 1.66–41.76). Adjusting the influence of colonizing pathogens for other factors has no effect on its statistical significance. Unlike the situation in patients with head and neck cancer, the presence of colonizing pathogens cannot be proved as statistically significantly associated with PEG site infection in neurological patients (Table 4). Moreover, adjusting the influence of colonizing pathogens for other factors presented has no effect on its insignificance. On the other hand, MRSA infection was found to influence the PEG site infection in patients with neurological disorders significantly (OR = 4.50, 95% CI: 1.08–18.76).

### 3.2. Mortality

30-day mortality after PEG placement was 20/274 (7.3%). Nineteen neurologic patients and 1 cancer patient died within 1 month after PEG insertion. In 12 patients, pneumonia was stated as the cause of death. No death was strictly related to PEG complication, but the presence of infection and the inability to use enteral feeding could contribute to the development of the pneumonia. No significant association was found between any factors (colonizing pathogens, resistant microorganisms, peristomal infection, indication, etc.) and mortality.

## 4. Discussion

During the pull method of the percutaneous endoscopic gastrostomy, the catheter is passed through the mouth, pharynx, and esophagus and may be contaminated by colonizing microorganisms. In this way, pathogenic microorganisms can be transported to the abdominal wall and, respectively, to the PEG wound.

An antibiotic prophylaxis had confirmed efficacy in the reduction of early infectious complications of PEG insertion [5, 8]. The spectrum of used antibiotics covers mostly Gram-positive strains and well-sensitive Gram-negative.

An increased prevalence of resistant strains like* Pseudomonas aeruginosa *and MRSA was observed from wound isolates of PEG patients with infectious complications [12].

Preprocedural bacterial screening for MRSA and decontamination and tailored antibiotic prophylaxis was suggested by some authors [13]. However, the prevalence of MRSA is probably more variable in different subgroups of patients [14, 15]. The prevalence of MRSA colonization was not emerging in our study. But we found difference in view of indication group. Eleven neurologic patients were colonized and MRSA was assessed as the cause of the wound infection in three (27%) of them. None of patients with cancer was colonized by MRSA.

Local antibiotic policy may differ due to different nosocomial microorganisms in each hospital [16]. A monitoring of the bacterial colonization of PEG patients may help to tailor appropriate prophylaxis [17]. A routine prescription of antibiotic prophylaxis may lead to an increased prevalence of resistant pathogens with subsequent clinical problems [18, 19]. A screening of microbial colonization and differences among patients with different indications could be applied for further antimicrobial strategy.

The evaluation of the peristomal infection in our study was partially subjective more often using clinical judgment than calculated score systems [20]. This factor might reduce the number of assigned PEG site infections than reported in other studies. However, the clinical impact of any peristomal infection depends more on clinical signs and the course of disease than on the theoretically calculated points. The infection has generally minor severity and the management is individually determined. Therefore, a positive index score may include more potential, rather than actually existing clinical problem.

The etiology of peristomal infection is complex. Infection in patients without pathogens may be due to local abdominal wall inflammation, suboptimal wound care, or individual contribution of skin colonizing agents to the development of infection.

The PEG site infection was significantly more common among patients with pathogens, independently on the antibiotic sensitivity in patients with head and neck cancer.

Our data demonstrates that the microbial agents colonizing oropharynx or airways are in significant concordance to the peristomal infection pathogens. The concordance rate was not so high as recently reported by Taiwan study (16 from 19 concordant) [21] and Japan (20/21 concordant) [22]. The main difference is probably in the high prevalence of pathogens in our cohort (69%), in patients with neurological disorders particularly.

Although no significant difference was observed in the incidence of the peristomal infection between patients with and without the pathogens in the oropharynx and airways, severe infections developed only in the patients colonized by the resistant pathogens. Our study further suggests that it is very difficult to tailor the appropriate antibiotic prophylaxis only by monitoring flora from oropharynx, airways, or PEG wounds.

The detection of multiple pathogens colonizing neurological patients is not a reliable basis for tailored prophylaxis. The increased prevalence of amoxicillin resistant pathogens is probably more due to the colonization during prolonged hospital stay without any important clinical relevance to the peristomal infection.

Many other risk factors including diabetes mellitus, malnutrition, inflammatory status, and low gastric acidity may potentiate the development of the peristomal infection [4, 23]. Our study showed no difference in development of the infection due to the serious comorbidities and tracheostomy. Our cohort of patients is more homogenous and selected due to exclusion of patients with signs of active inflammation and infection before PEG procedure.

Some authors found that the patients with malignancies are at greater risk for the peristomal infections than those with nonmalignant diseases [24]. This finding might be supported by our data, which indicated a statistically nonsignificant trend towards development of the PEG site infection in cancer patients but a highly significant association between the presence of oropharyngeal pathogens and the incidence of the infection in that subgroup of patients.

The previously published results on a small group of patients, stating that cancer patients colonized by pathogens are at increased risk of the peristomal infection [25], could be supported by our findings.

The detection of pathogenic microorganisms in oropharynx prior to PEG placement could be a tool for screening high risk oncological patients for the development of an infectious peristomal complication. It might be beneficial to consider waiting for microbiological swab results for 2-3 days prior to PEG placement. Targeted antibiotic prophylaxis for an even longer time (i.e., 24–48 hours) [26] and particular wound care using specific local disinfectant [27] might be appropriate precautions to decrease the risk of a wound infection in such patients. Also, new antibiotic prophylactic strategy using oral cotrimoxazole [6] may be effective and probably safer for patients than prolonged untailored prescription of broad spectrum antibiotics.

The main goal of our study was to determine the utility of oropharyngeal bacterial screening in patients indicated for PEG. The study showed that oropharyngeal microbial colonization is not the crucial factor for PEG site infection development but it may be an easy indicator. The increased number of infection in cancer patients may by probably attributed to poor hygiene of oral cavity, worse nutrition, and some alteration of immunity due to oncological disease and its therapy. There is no specific pathogen in the cancer patients that causes the PEG site infection. In view of patients with head and neck cancer we can conclude that oropharyngeal colonization with pathogens is a general marker of increased risk of infectious complications only.

In neurological patients the screening for pathogens was not found to be helpful in our study. An exception to this would be the detection of MRSA in long-term institutionalized patients, depending on actual microbiologic surveillance in the hospital.

## 5. Conclusions

The impact of oropharyngeal bacterial screening prior to percutaneous endoscopic gastrostomy is different among oncological and neurological patients.

The detection of pathogens may be a marker of the increased risk of the peristomal infection in cancer patients only. Such patients may require particular care of the wound and prolonged antibiotic administration. In neurological patients the benefit of the screening is limited to the detection of MRSA carriers. Subsequent tailored prophylaxis may be applied.

## Figures and Tables

**Table 1:** Summary of patient characteristics according to indication for PEG (*n* = 274).

Variable	Neurological disorders (*n* = 206)		Head and neck cancer (*n* = 68)		*P*
	*n*	%	*n*	%	
Sex (male)	116	56.3%	45	66.2%	0.159
Colonizing pathogens	159	77.2%	31	45.6%	<0.001∗
Polymicrobial flora	75	36.4%	12	17.6%	0.004∗
Pathogens resistant to amoxicillin	93	45.1%	12	17.6%	<0.001∗
Tracheostomy	67	32.5%	13	19.1%	0.045∗
MRSA infection	11	5.3%	0	0.0%	0.071
ATB prophylaxis	206	100.0%	68	100.0%	1.000
Coamoxicillin	196	95.1%	66	97.1%	0.736
Cefuroxim	3	1.5%	1	1.5%	1.000
Other	7	3.4%	1	1.5%	0.684
Serious comorbidities	32	15.5%	7	10.3%	0.324
PEG site infections	18	8.7%	12	17.6%	0.070
Other complications	17	8.3%	12	17.6%	0.040∗
Major complications (bleeding, respiratory)	5	2.4%	6	8.8%	0.027∗
Minor complications	12	5.8%	6	8.8%	0.394
30 days of mortality	19	9.2%	1	1.5%	0.032∗

*Statistically significant at 5% level.

**Table 2:** Pathogenic microbial agents colonizing oropharynx or airways in PEG patients and agents isolated from PEG wound in patients with clinically apparent infection.

	Colonizing	Agents	PEG wound infection
	All 274 pts	Neurological/cancer (206/68 pts)	Neurological/cancer (18/12 pts)
*Ps. Aeruginosa * **(R)**	63	61/2	1/0
*Candida* sp. **(R)**	23	16/7	2/3
**MRSA (R)**	**11**	**11/0**	**3/0**
*Klebsiella* sp. ESBL **(R)**	9	9/0	2/1
*Enterobacter* sp. **(R)**	4	2/2	2/1
*Klebsiella* sp. (S)	33	25/8	7/0
MSSA (S)	26	20/6	4/5
*Citrobacter* sp. (R)	2	1/1	0/0
*Proteusmirabilis* (S)	14	12/2	3/1
*Acinetobacter* sp. (S)	10	6/4	0/0
*E*. *coli* (S)	12	9/3	1/1
*Enterococcus faecalis* (S)	6	6/0	0/1
Streptococ beta hemolytic (S)	6	0/6	2/2
*Polymicrobial *	*86 *	*75/11 *	*14/10 *

(R): amoxicillin resistant (S) amoxicillin sensitive, MRSA: methicillin resistant *Staphylococcus aureus*, MSSA: methicillin sensitive *Staphylococcus aureus*.

**Table 3:** Characteristic of patients with PEG site infection (*N* = 30).

Indication	All	Neurological	Cancer
PEG site infection	30 (10.9%)	18 (8.7%)	12 (17.6%)
*Severe infection *	* 5 (1.8%) *	* 5 (2.4%) *	* 0 *
Concordance between oropharyngeal and PEG site pathogens	17 (57%)	10 (56%)	7 (58%)
ATB prophylaxis	30 (100%)	18 (100%)	12 (100%)
Coamoxicillin	29 (97%)	17 (94%)	12 (100%)
Cefuroxime	0	0	0
Other	1 (3%)	1 (6%)	0
30 days of mortality	4 (13%)	4 (22%)	0

**Table 4:** Univariate analysis of possible risk factors with respect to the PEG site infection in all patients and separately for head and neck cancer patients and neurological patients. *Statistically significant at 5% level.

Variable	All (*n* = 274)			Cancer (*n* = 68)			Neurological (*n* = 206)		
	OR	95% CI	*P*	OR	95% CI	*P*	OR	95% CI	*P*
Sex (male)	1.49	0.65–3.39	0.330	1.03	0.27–3.85	0.969	0.46	0.17–1.24	0.126
PEG indication (cancer)	2.24	0.95–5.26	0.070		n.a.			n.a.	
Colonizing pathogens	1.52	0.59–4.07	0.408	8.33	1.66–41.76	0.010∗	0.75	0.25–2.22	0.601
Polymicrobial flora	0.76	0.30–1.90	0.678	3.00	0.73–12.34	0.128	0.47	0.15–1.49	0.199
Pathogens resistant to amoxicillin	0.92	0.39–2.15	1.000	1.74	0.39–7.71	0.466	0.97	0.37–2.57	0.950
MRSA colonization	3.28	0.64–14.80	0.107		n.a.		4.50	1.08–18.76	0.039∗
Serious comorbidities	1.24	0.39–3.71	0.781	0.76	0.08–6.94	0.806	1.63	0.50–5.32	0.416
Tracheostomy	0.87	0.34–2.17	0.834	1.53	0.35–6.70	0.570	0.78	0.27–2.29	0.653
Other complications	0.93	0.21–3.54	1.000	0.37	0.04–3.19	0.367	1.44	0.30–6.87	0.646

n.a.: cannot be evaluated.
